# Supplementary material for: Public perceptions on carbon removal from focus groups in 22 countries
Source: Nat Commun. 2024 Apr 24;15:3453. doi: 10.1038/s41467-024-47853-w (PMC11043362; doi:10.1038/s41467-024-47853-w)
Supplement: Supplementary file 1 — Supplementary Information [file 41467_2024_47853_MOESM1_ESM.pdf]

SUPPLEMENTARY INFORMATION

Public perceptions on carbon removal from focus groups in 22 countries

Sean Low[1*], Livia Fritz[1], Chad M. Baum[1], Benjamin K. Sovacool[1,2,3]

[1] Department of Business Development and Technology, Aarhus University, Birk Centerpark 15, 7400 Herning, Denmark
[2] Science Policy Research Unit (SPRU), University of Sussex Business School, Jubilee Building, Arts Rd, Falmer, Brighton BN1 9SL, United Kingdom
[3] Department of Earth and Environment, Boston University, 685 Commonwealth Ave, Boston, MA 02215, United States
* Correspondence and requests for materials should be addressed to Sean Low (email: sean.low@btech.au.dk).

Supplementary Table 1 | Carbon removal approaches

**Afforestation and reforestation (expanded into restoring and/or growing vegetation)**<br>**Complexes: Hopes, concerns, governance**		**National focus groups mentions**<br><br>**North (11 countries)**<br>**Emerging South (8 countries)**<br>**Developing South (3 countries)**<br><br>**R=Rural focus group**<br>**U=Urban focus group**	**Representative quotes**
**Accelerating distributed and diverse practices**	Hopes: Naturalism, Known practices, Agency	**All groups (22/22)**	**Austria, Rural:** … it changes nature back to the way it was before we started affecting it… I think it's an ideal accompanying measure. And above all, it's more manageable technologically (than direct air capture). So to every regular person, this seems less abstract than the other approaches.<br><br>**Indonesia, Urban:**… these trees serve such functions, spiritually and also in real life. Even though this method is not as fast as other methods to lower the temperature, in the long term, it has a better impact… Maybe it could lessen famine in society, by selecting trees that provide a certain fruit. Global warming… causes drought as well, and drought decreases natural and food resources.
	Governance: Ban or disincentive deforestation	**North (4/11)**: Austria R, Norway U, Spain U, Switzerland R<br><br>**Emerging South (2/8):** Indonesia R, South Africa R<br><br>**Developing South (3/3)**: Dominican Republic R, Kenya R, Nigeria U	**Kenya, Rural:** People with money tend to live next to rivers and construct their homes near the rivers and encroach on the wetland. If the government can regulate that and put strict laws discouraging them from living in those areas … I think we need to campaign for people that are cutting down trees and we need to campaign for people regrowing more trees. I think our government should look at more opportunities for creating things like jobs … because people tend to cut down trees in search of better living, so they can sell it to the market.
	Governance: Diverse ecosystems-use, Public-private initiatives	**North (8/11)**: Australia R+U, Germany U, Italy R+U, Norway R+U, Poland U, Spain R+U, Switzerland R, USA R<br><br>**Emerging South (3/8):** Brazil U, Indonesia U, South Africa R	**Brazil, Urban:** Here in Rio de Janeiro, in the western part, there's a mangrove where some NGOs, the city hall and some companies have projects to conserve this area. Some green areas here in the south part are also preserved by local commerce and in return, the government reduces some taxes… this makes other companies engaged and this became a chain, regional, national, and global and it grows larger.<br><br>**India, Urban:** Agricultural institutes would have academic interest in this, as well as NGOs, who would be useful because they are used to communicating with people, especially in a country with so many regional languages. And I think you should also

		**Developing South (3/3)**: Dominican Republic R, Kenya R+U, Nigeria U	include the start-ups… who already are in sync with the rural population, bringing in new technologies, ways of doing production and all… I think it is a readymade thing for us.
	Governance: Urban opportunities	**North (6/11)**: Austria U, Italy R, Norway U, Spain R, Sweden U, USA R<br><br>**Emerging South (2/8)**: China R, India R<br><br>**Developing South (2/3)**: Nigeria U, Kenya R	**Norway, Urban:** Where can it be placed… does it matter where they are? Bike lanes or the centre lane on roads, or is it croplands. Who owns the earth where they're placed; do they have permission to place them? Should there be many or just a few random that are placed out? Can they be put on roofs? There's a lot of research now, they're putting a lot of earth and grass on roofs, can it be placed there? It's very exciting, you might not need to use cultivated land.
**Spatial trade-offs: Carbon stocks vs. food, residence, context of deforestation, corrupt governance, and business pressures**	Concern: Spatial trade-off, Food crops	**North (8/11)**: Australia R+U, Austria U, Germany R+U, Italy R+U, Norway U, Poland U, Spain U, Switzerland U<br><br>**Emerging South (3/8)**: Brazil U, China R+U, India R+U<br><br>**Developing South (2/3)**: Kenya R+U, Nigeria R	**Nigeria, Rural:** The only problem is that… if we need so much space – because the amount of CO_2 we are talking about is not a small one – so that means we need land to plant trees to absorb the CO_2, just like where we talk about the land to plant bioenergy crops… with time we will run out of food. There would be food shortages because we would need land for the vegetation.
	Concern: Spatial trade-off, Residential development	**North (7/11)**: Australia U, Germany R, Italy R, Norway R, Poland U, Sweden U, USA R<br><br>**Emerging South (5/8)**: Brazil U, China R, India R+U, Indonesia U, South Africa R<br><br>**Developing South (1/3)**: Kenya R+U	**Indonesia, Urban:** It requires a lot of land to do these things: reforesting, replanting vegetations and such. In Indonesia, the population increases significantly. So, there's this question of, like, do we have the space/land area to do this? A vacant space that we could use … would it sacrifice buildings where we live? Would it sacrifice the development for the people? Would the government be able to provide a replacement/resettlement? Would the government be able to provide compensation?
	Concern: Implications of spatial trade-off, Property seizure	**North (2/11)**: Australia R, USA U<br><br>**Emerging South (2/8)**: China R, India U<br><br>**Developing South (1/3)**: Kenya U	**India, Urban:** You know, there is a risk regarding where (afforestation) should be done and what type of land they choose: if the chosen land is already cultivated and the destruction (sic) will impact the lives of the people who are living there. Whether in urban or rural areas, you see landowners will already have different practices.

	Concern: Impermanence, profit motives	**North (5/11)**: Austria U, Germany U, Norway U, Poland R, Spain U<br><br>**Emerging South (2/8)**: Chile U, Indonesia R<br><br>**Developing South (1/3)**: Kenya R+U	**Chile, Urban: …** if we talk about reforestation, (companies will plant) 1,000 pines in the south of Chile and then cut them down after 5 years to make lumber. Just don't allow the big companies to join this project because they are going to do the reforestation and they are going to cut them down. We would have to verify specifically what the reforestation would be and in what aspect.<br><br>**Germany, Urban:** … we cannot just plant a tree or build a sea grass plantation, we need to protect them for the next 30-40 years. If I buy a plane ticket, I can donate something for planting a tree, but I do not know if it will still be there in two years.
	Concern: Marine carbon removal and capacity to reduce land-use trade-offs	**North (4/11)**: Australia U, Germany U, Norway U, UK R+U	**Norway, Urban:** … we see how vulnerable we are when it comes to agriculture, and we're dependent on that. We've also talked about how it can be stored in seaweed, in seaweed forests in the ocean… when it's in the ocean… as long as it doesn't affect the fish stock and fish life, it might be better to use (marine systems)… but... I need facts, weigh the pros and cons.
	Analogy: Amazon deforestation	**North (5/11)**: Australia U, Austria R+U, Germany R, Norway, U, Switzerland R<br><br>**Emerging South (1/8):** Brazil R+U	**Brazil, Urban:** I can only see benefits in all those ideas. I think that here in Brazil we work with all these solutions already. Perhaps one of the things we could do is to suggest that every country, especially the developed ones, adopt the Brazilian Forest Code, but they wouldn't accept it.<br><br>**Norway, Urban:** It's important to focus there, because that is the lung of the Earth with the forest there. And it's being cut down, and if there's one place that needs protection on the planet, it's that whole continent there. A government (at time of discussion, Bolsonaro) is needed there to crack down on it, but it doesn't seem like they're that interested in it because they probably make more money selling it.
**Soil carbon sequestration (including but not interchangeable with biochar)**<br>**Complexes: Hopes, concerns, governance**		**National focus group mentions**	**Representative quotes**
**Accelerating distributed and diverse practices**	Hopes: Naturalism, Known practices, Agency	**All groups (22/22)**	**Norway, Rural:** I love this, I think it's genius. Here, I thought that this is a typical strategy that the political parties can go to election with… As long as the person owning the land is a willing participant, it shouldn't be any problems if a county or municipality is asking that it's done … Then I also think it's important in many parts of the country to use those who know the earth, who know the vegetation. Like indigenous people, farmers, who have carried on traditions for hundreds of years… That can start a ripple effect and make it more accepted, or more the norm to do it that way.

	Hope: Known practices, Alternatives to importing artificial fertilizer, including biochar as substitute for pesticides	**North (2/11)**: Italy R, Norway U<br><br>**Emerging South (1/8)**: Brazil R+U<br><br>**Developing South (2/3)**: Dominican Republic U, Kenya R	**Dominican Republic, Urban:** Well, this is a great substitute for pesticides and herbicides. For example, coffee and cacao, which are the main products of the country, it could help the plantation owner to save money and help the weather at the same time since we wouldn't get poisoned by its chemicals, something quite common with pesticides and weed-killers.
	Governance: Education and consultation, Economic incentivization	**North (7/11)**: Australia R+U, Germany R, Poland R, Spain U, Norway R+U, UK R, USA U<br><br>**Emerging South (7/8)**: Brazil R+U, India U, Indonesia U, Saudi Arabia R+U, South Africa U, Turkey R,<br><br>**Developing South (2/3)**: Kenya R, Nigeria R+U	**Turkey, Rural:** It is important both for the satisfaction of the society and for it to become a more direct democracy. As I said, we should not think of farmers like a farmer sitting in his tiny garden. It can also be an industrial farmer. It varies by region. The goal here is for everyone to express their opinion. Since there will be a vote, scientists will be there too. Notifications are made. This is an issue that can be developed. But farmers are also an important mass in terms of legitimizing the idea.
**Agricultural and food security**	Hope: Preserve or enhance agricultural capacity	**North (10/11)**: Australia U, Austria U, Germany R+U, Italy R, Norway R+U, Poland R+U, Spain R, Sweden R, UK U, USA U<br><br>**Emerging South (7/8)**: Brazil R+U, India R+U, Indonesia U, Saudi Arabia R+U, South Africa R, Turkey R<br><br>**Developing South (3/3)**: Dominican Republic R+U, Kenya R+U, Nigeria R+U	**Dominican Republic, Rural:** Well, that has an impact on the economy because there is more food production and I also believe that a country with food production has less scarcity, so it helps in every sense of the word.<br><br>**Spain, Rural:** This could be positive, as long as it is controlled, so they don't replace food crops with this kind of crops, food farming shouldn't be abandoned because it would make it very expensive.
**Direct air capture and carbon storage**<br>**Complexes: Hopes, concerns, governance**		**National focus group mentions**	**Representative quotes**
**Governance of DACCS as high-energy, high-cost systems**	Hope: Envisioned benefit for innovators and manufacturers	**North (11/11)**: Australia R+U, Austria U, Italy R+U, Germany U, Norway R+U, Poland R, Spain R, Sweden R, Switzerland R+U, UK U, US U	**Switzerland, Urban:** Finally, the manufacturers of the filter systems… they can make a big business out of it… with the storage facilities, would certainly also be interesting for business and industry, because ultimately it will pay off again if these products are promising and if they can be produced and sold. It would certainly be desirable for big players to get involved… individual companies could also make a

		**Emerging South (7/8)**: Brazil R+U, Chile U, India U, Indonesia U, South Africa R+U, Saudi Arabia U, Turkey R+U<br><br>**Developing South (1/3)**: Kenya R	valuable contribution… Although even in 'civilised' countries the state should control everything. Otherwise, I fear that once again the profit of the individual company will be in the foreground.
	Concern: negative perspectives on government-industry complex	**North (4/11)**: Australia R+U, Austria U, Italy R, Switzerland R+U<br><br>**Emerging South (3/8)**: Brazil U, Indonesia R, Turkey U<br><br>**Developing South (1/3)**: Dominican Republic U	**Australia, Rural:** It's totally unproven, this carbon capture and storage. It's easy enough to talk because there's plenty of money that goes to from politicians to their mates, and it goes to energy companies.
	Concern: technological and financial capacities and burdens	**North (6/11)**: Australia R, Germany R, Italy R, Poland U, Switzerland R+U, Sweden U<br><br>**Emerging South (3/8)**: Brazil R, India R+U, South Africa U, Saudi Arabia U<br><br>**Developing South (2/3)**: Dominican Republic R, Kenya U	**Kenya, Urban:** It might be a challenge especially to this country or all of the third world countries whereby you might end up spending a lot of money… I don't expect my government to come in and impose (sic) the technology whereby they're capturing the carbon (without) using a bigger percentage of the taxpayers' money to impose this technology. I'm a horticultural farmer by nature. I don't expect the government to come and tell me we are building this technology, when we don't even have roads to transport whatever I have from my farm.
	Governance: Unequal technological capacities, convergence on tech-transfer	**North (3/11)**: Italy R, Poland R, Switzerland R+U, Sweden U<br><br>**Emerging South (3/8)**: Brazil R, India U, South Africa U<br><br>**Developing South (1/3)**: Kenya U	**Switzerland, Urban:** The industrialised countries have a special responsibility because we also cause higher CO2 emissions and would also have the economic possibilities to implement such projects, which cannot be expected from a country that does not have the means to do so, or at least not now.<br><br>**Brazil, Rural:** Obviously, not all the countries have big companies that can do that – for example, poorer countries. Nonetheless the richest countries could invest so that these ideas could reach these other countries.<br><br>**Poland, Rural:** Countries that can't afford it should get some huge subsidies. Let these countries of the third world have access to this, because the chances should be adjusted.

**Concerns and governance of hazardous siting, leakage, and pollution exports**	Concerns: NIMBYism	**North (5/11)**: Austria R+U, Germany R+U, Norway R+U, Spain R, Switzerland R, UK R<br><br>**Emerging South (1/8)**: Chile U	**Norway, Rural:**… everyone's against having these big installations near them, but that's also because we don't see the long-term consequences. And then we don't want the downsides of cleaning up before the future generations come. So then you get the 'windmill syndrome'. We don't want to see it, but we want it.
	Concerns: Hazardous siting, Leakage	**North (10/11)**: Australia R+U, Austria R+U, Germany R+U, Italy R+U, Poland R+U, Spain R, Sweden R+U, Switzerland R, UK R+U, USA R+U<br><br>**Emerging South (4/8)**: Brazil U, China R+U, India R, Turkey R+U,<br><br>**Developing South (2/3)**: Kenya U, Nigeria U	**Kenya, Urban:** Suppose the liquid itself leaks… In the third world countries, most of the countries we depend on farming, we don't know the impact that will be caused by this liquid carbon dioxide, carbon dioxide in the soil and for agriculture we depend on the soil, so the most people to be negatively affected by this. Of course, these will be the farmers.
	Concern: Sabotage	**North (1/11)**: USA U<br><br>**Developing South (1/3)**: Nigeria U	**Nigeria, Urban:** When I was in Niger State, our… (pipe)line hardly could go through because of the insecurity and pipeline vandalization… imagine the blast that happened in a community, it wrecked the whole community – and this is gas. You never can tell if it is tampered with, what the ripple effect is.<br><br>**USA, Urban:** I guess I would be concerned because it says that you need pipelines to transport the carbon and underground places to store it. So if you're storing it, who's watching it, or who is monitoring what's already what's there.

	Pollution exports	**North (6/11)**: Australia U, Austria R+U, Germany U, Poland U, Sweden U, Switzerland R<br><br>**Emerging South(1/8)**: Brazil R	**Australia, Urban:** We were already using underground dumps for storing things like nuclear and other biohazards. And then… countries are going to be upping the ante for this kind of thing because you've already got very poor countries accepting waste from other countries.<br><br>**Austria, Rural:** And of course, the location of the storage is problematic… in this case we are considering something which is meant to benefit the whole world. You're not doing something directly for your country, so no one wants to take on the drawbacks because everyone will benefit from the advantages… Of course, you could do it in a way that states agree that they each get money from the overall global pot which they can use at a national level…. I think it could be done in this way, especially in areas – perhaps in Africa – where due to the heat there is little agricultural potential for the land, and in return for building these structures the population which lives there is rewarded with money, and in that way you can revive the region. We're talking about huge installations on these big areas, so it has to benefit the people who live there – you can't just build it.
**Governance: Polluter pays**	Concern: Energy efficiency, disguising and prolonging fossil energy	**North (8/11)**: Australia R, Austria R, Italy U, Poland R, Norway U, Poland U, Sweden R, UK U<br><br>**Emerging South (3/8)**: China U, India R, Turkey R<br><br>**Developing South (1/3)**: Nigeria R	**Poland, Urban:** … in the case of direct air capture we are talking about gigantic plants, pipelines, energy that will required for transporting and storage, and it's (analogous to) electric cars and transport. Well, cool – everyone gets excited about electric cars, except that electric cars in Poland run on electricity generated by power plants 90% of which run on coal.
	Governance: Polluting industries should pay	**North (6/11)**: Australia R+U, Poland R, Spain R, Sweden R+U, Switzerland R, UK U<br><br>**Emerging South (4/8)**: Brazil R+U, China U, India U, Saudi Arabia R	**Brazil, Rural:** those companies need to be bound to reverse the pollution process. If they are alone, they could choose either to do something or not, but if the government decides to create a plan for the companies that pollute to also be the ones responsible for doing something to reverse the scenario, it would be more likely to happen. I think it's a matter of government and companies.
	Governance: Oil and gas	**North (4/11)**: Australia U, Austria U, Spain R, UK U	**UK, Urban:** The flip side for these is I suppose, if you're currently capturing no CO2 from the energy and gas production, and you need to use, say 20% of the total energy used to do the capturing, then you'll just have to capture 120% of the CO2 and that

			cancels out. I know that's probably a lot of money but you can make the oil and gas companies pay for it…. You know when you get water in your house you have to pay for it to come out the tap, you also have to pay for it to go down the drain.
	Governance: Industry should take initiative, Corporate resources and social responsibility	**Emerging South (2/8)**: China R+U, India R+U<br><br>**Developing South (1/3)**: Dominican Republic U	**China, Urban:** As long as large enterprises are willing to make contributions to environmental development, have such funds and resources and scientific research capability, they can directly seal up the carbon generated from their own factories or locations and put then underground. As long as they can do it themselves and promote this idea on a large scale. Maybe it needs the government to do publicity or policy about this request.
**Enhanced weathering**<br>**Complexes: Hopes, concerns, governance**		**National focus group mentions**	**Representative quotes**
**Governance depends on sense-making of EW through DACCS (reliant on mining/energy industry)**	Concern: Expanding mining operations, spatial trade-offs, land-grabs, hazardous siting	**North (5/11)**: Austria R, Italy R+U, Sweden R+U, UK R, USA R<br><br>**Emerging South (1/8)**: Brazil R<br><br>**Developing South (2/3)**: Kenya R, Nigeria R	**Austria, Rural:** Here I see a huge interference with nature, because I assume you'd need a large amount of stones for this, and if you have to blast whole mountains in order to get the stones to capture CO_2 and then bring them to somewhere that they usually wouldn't ever get to.<br><br>**Italy, Urban:** The point is that we have to manufacture it, and in order to manufacture it they need energy. The one needed to break the stones as well, is it a sustainable energy or is it producing carbon dioxide? Are we back to the start?<br><br>**Nigeria, Rural:** Like we said, land owners should be considered, like we said the rocks would not be found everywhere, there are certain places it can be found which belong to someone in the rural area… There should be policy against illegal mining, there should be laws and rules guiding them…our recourses should be used rightly.
	Hope: Recycling mining waste	**North (1/11)**: Australia R+U<br><br>**Emerging South (2/8)**: Chile U, South Africa R	**South Africa, Rural:** We do have the mines with large volumes of rocks coming out of the mines and they don't know where to dump them. Most probably we should be cleaning up those quarry stuff. No littering of rocks all over the country…. I think we should recycle available wastes or rocks and play with that. But as for going to blow up completely new rocks? I'm not for that.
**Governance depends on sense-making of EW through biogenic**	Concern: Property seizure, land grabs	**North(2/11)**: Sweden U, US U<br><br>**Developing South(1/3)**: Kenya U	**US, Urban:** … what kinds of areas can be used? What beaches do they have to have? You don't want to impact the public beaches… It's got to be something that's safe for people to be around. Yeah. And farmlands… I do think that too many, too many farm lands being bought up by the big companies to build those huge businesses, and we need more of our farm lands, because that's where we can help do this kind of work.

**approaches (naturalism)**	Hope: EW as soil enhancement, naturalism and known practice	**North (2/11)**: Italy R, Germany R<br><br>**Emerging South (2/8)**: India U, Indonesia U<br><br>**Developing South (1/3)**: Nigeria U	**Nigeria, Urban:** I think they have to put all these into practise. Because telling a local farmer that if you break a rock, it will enhance soil and farm produce… I think they should practicalize and let them see. Actually in local areas we don't believe in chemical manure, we believe in natural manure… I should know how it works first. I think samples should be carried out, they should identify the type of rock that would be needed.
**Bioenergy carbon capture and storage**<br>**Complexes: Hopes, concerns, governance**		**National focus groups mentions**	**Representative quotes**
**Accelerate known practices in bioenergy**	Hopes: Bioenergy and biofuel sector, Less carbon intensive fuel, Energy security	**North (6/11)**: Australia R, Italy U, Norway U, Spain R, Poland R, USA U<br><br>**Emerging South (1/8)**: Brazil U	**Australia, Rural:** I could see a benefit in aid with bioenergy with carbon capture and storage, because it's probably going to lower pollution. So it's going to solve one problem there… I think transport, like the bio energy, if that was a fuel source for cars and industry and an ultimate one, I think that it would make us more secure as well as self-sufficient. So Australia or any country wouldn't be relying on other countries for oil, and, you know, those kinds of fossil fuels.<br><br>**US Urban:** So I think this could be more of energy companies, because that could be a way for them to change their profit structure, and use this as their way to make money. Because they're in the process, they're in the business of providing the power. So this is something that they might consider, just like I said, my utility company uses solar and nuclear power, to lessen the cost from oil.
	Governance: Expand known practices		
**Mitigating known trade-offs from bioenergy conflicts**	Concern: Spatial trade-offs, Mirror known food vs. fuel conflicts	**North (2/11)**: Australia U, Austria U<br><br>**Emerging South (1/8)**: India U<br><br>**Developing South (3/3)**: Dominican Republic R, Nigeria U, Kenya U	**Austria, Urban:** How should it be different to biodiesel, which had countless negative side effects? The EU subsidised it; entire forests were cut down and instead olive trees were planted. In principle it's the same. Plants also absorb CO2 from the atmosphere. It creates emissions and blasts them back into the atmosphere. It was terrible. I wouldn't want that at all.<br><br>**India, Urban:** Food industry will be affected more. Because the farmers would be thinking that growing these kinds of plants is beneficial, we shift from food crops to CO2 plants, so that the food industry will also be affected in the future. So, we would face some food scarcity also.
	Governance: Waste crops, abandoned land	**North (1/11)**: US U<br><br>**Emerging South (1/8)**: Brazil U	**US, Urban:** But there is, I think there's a lot of land that maybe abandoned, or places where this could be implemented without asking farmers to donate their land for that. And I'm not saying oh, we have to take up everything that's free. But maybe we should take some farmland in some forest or some urban areas or suburban areas. I

			think even where I live where there are in a city environment, there's a lot of abandoned homes, where that land could be used for that, and still be close to a general population.
**Analogous concerns with DACCS**	Concern: Hazardous siting and leakage, Polluter pays	**North (3/8)**: Australia U, Germany R, UK R, Switzerland R<br><br>**Emerging South (1/8)**: South Africa U	**UK, Rural:** Because if you're burning crops, processing the smoke and stuff like that, would it have an effect on the towns near them? Would there be a smell? Would there be any emissions or fumes? I'm not really sure, because it sounds like they would obviously filter and catch everything that comes from the fumes of the smoke … But I've read about storing carbon underground a while ago, and I'm not a hundred percent behind it because you would need to build reservoirs or some storage containment.<br><br>**Switzerland, Rural:** It should be financed by the huge profits that are being made by the oil companies, which are actually the cause of the whole dilemma. For me it is incomprehensible that I suddenly have to pay twice as much, or even more than twice as much, for my wood heating as I had to pay two years ago. And all because of the oil and gas prices, which have risen by thirty percent.
**Misconceptions**	Misconception: Double removal	**North (1/11)**: Poland U<br><br>**Emerging South (4/8)**: Brazil U, Chile U, China U, India U	**Brazil, Urban:** BECCS is interesting because it makes a double removal of the carbon dioxide from the air. The plants capture this carbon dioxide and then they are cut to generate fuel for technologies that also remove carbon dioxide, so it's a double removal, it has better efficacy in less time.

Complexes of perspectives are marked by black bolded lines, linking hopes, concerns, and governance. Supplementary Table 1 is a longer version of Table 1 (in text), with all themes (hopes, concerns and governance), as well as additional quotations from a wider range of focus groups. Here is also included (in the column 'National focus groups mentions') a count of the focus groups and countries that spoke to a certain theme.

Supplementary Table 2 | Climate and sustainability action

**Dimensions**		**National focus groups mentions**<br><br>**North (11 countries)**<br>**Emerging South (8 countries)**<br>**Developing South (3 countries)**<br><br>**R=Rural focus group**<br>**U=Urban focus group**	**Representative quotes**
Carbon removal as a supplement within climate and sustainability action	Carbon removal does not address underlying causes of climate change	**North (4/11)**: Austria R, Italy R, Norway U, US R<br><br>**Emerging South (3/8):** China U, South Africa U, Turkey U	**China, Urban:** We need solutions which address the underlying/fundamental problem. The environmental problem was caused by this generation – excessive use of energy, rapid industrialisation, uses of gasoline cars, air conditioning…all things which have affected the environment negatively. If the underlying problem is not addressed, the negative impacts will be passed to our sons and grandsons (future generations). We need to be committed to solve the underlying problem.
	Integrate carbon removal into land-use, agriculture and forestry	**North (2/11)**: Germany U, Spain R<br><br>**Emerging South (1/8):** Brazil U<br><br>**Developing South (1/3)**: Kenya	**Spain, Rural:** We should try and avoid intensive farming. We should probably go back in time to an extensive farming, taking care of land, nature, and mountains. We should try and embrace agriculture again, because we're a country that could have amazing farming, and we depend on the sunflower coming from Ukraine, that's insane.<br><br>**Brazil, Urban:** But in reality, there are a lot of other points that can't be ignored when talking about this. For example, deforestation in order to create a space for cattle. Cattle are responsible for 50% of the emission of carbon dioxide and there's also methane and nitro dioxide are also produced by cattle.
Buying time vs. Delay	Buying time for decarbonization; Engineered carbon removal buys time for restoring natural landscapes	**North (3/11)**: Australia R, Italy U, UK U<br><br>**Emerging South (2/8):** China U, South Africa U	**UK, Urban:** I think anyone, everyone benefits and the reality of the situation is that to transition the entire world onto renewable energy is going to take a while. There's still quite a long way to go. And I think everyone agrees that that's where we've got to be eventually bit that's going to take a while and I think we have to tackle climate change now, before we tackle that transition which means while there's still energy being produced by oil and gas and fossil fuels, that energy has to be captured, otherwise it just goes into the atmosphere. Everyone benefits because it's working towards the survival of the human race.

	Mitigation deterrence	**North (6/11)**: Australia R, Austria R, Germany U, Spain R, Sweden R, UK R<br><br>**Emerging South (1/8):** Brazil R<br><br>**Developing South (1/3)**: Dominican Republic U	**Austria, Rural:** With regards to the risks, I do see that if we sequester the CO_2 we always have the side-effect, in communications with citizens of the earth, that their behaviour does not change because they believe that it is a solution to the excess production of CO_2. I think that's the biggest risk. If we were to sequester it through technology, then the thoughts will continue that we can allow everything to carry on as it is now because it will be captured in another way. I think we need to be very sensitive to that in our communication and only present is as a complementary solution and not a panacea for the problem that we have.<br><br>**Dominican Republic, Urban:** Well, it would take a problem out of their minds, also for businessmen who still don't want to change from fossil fuels to something more eco-friendly. For example, we are trying to change everything to solar panels, electric cars, but since it's not that beneficial for corporations there are some restrictions.

Supplementary Table 2 is a longer version of Table 2 (in text), with all themes (hopes, concerns and governance), as well as additional quotations from a wider range of focus groups. Here is also included (in the column ‘National focus groups mentions’), a count of the focus groups and countries that spoke to a certain theme.

Supplementary Table 3 | Cross-cutting governance dimensions

**Assessment**<br>**Governance dimensions**	**National focus groups mentions**<br><br>**North (11 countries)**<br>**Emerging South (8 countries)**<br>**Developing South (3 countries)**<br><br>**R=Rural focus group**<br>**U=Urban focus group**	**Representative quotes**
Role: Triple helix - Universities, industry, government	Evidence-base for decision-making: **All groups (22/22)**<br><br>Triple helix:<br>**North (2/11)**: Norway U, US U<br><br>**Emerging South (3/8):** Brazil R, Indonesia U, South Africa R<br><br>**Developing South (1/3)**: Dominican Republic R	**Dominican Republic, Rural:** The government could contribute to the universities, then, the universities teach entrepreneurs, and then businessmen campaigning to raise awareness among others, hence, everyone is learning and contributing something that detriments climate change yet benefits the environment.<br><br>**Indonesia, Urban:** Maybe these scientists, the people from educational institutions, they'd conduct research to know whether it could go on/run or not. And then, the economical side. Like, those economists would research about the costs in the future as well. Secondly, it's the government. Maybe they're more about the policy. Like, they're going to set the rules/regulations and such. Also, the socialization (meaning: information and consultation) of those things to the people.
Actors: National scientific bodies, government agencies, ministries	**North (6/11)**: Australia R+U, Italy R, Poland R, Sweden R, Spain U, USA R<br><br>**Emerging South (5/8):** China U, India U, Indonesia R+U, Saudi Arabia R+U, Turkey U<br><br>**Developing South (3/3)**: Dominican Republic R, Kenya R, Nigeria U	**Australia, Urban:** Well, I think it would have to be publicly funded initially unless you had sort of some buy-in from the corporate sector. I mean, at some point, they're going to have to weigh in anyway. (An appropriate institute might be) the Commonwealth Scientific Industrial Research Organization (CSIRO). The point I'm making is that practically every country has its own version of CSIRO.<br><br>**India, Urban:** I think for me the big government organisations like coal authority of India, ONGC or other government sectors, because they allot the beds for the mining…
Rationale: Mistrust of leading governmental or industry involvement	**North (5/11)**: Australia R+U, Germany U, Italy U, Poland R+U, Spain U, Switzerland U	**Australia, Urban:** I think there's plenty of evidence to show that scientists that are funded from the private sector are encouraged to skew their data towards whoever's paying their bills. It needs to be independent funding. It cannot be funding based on any of the companies that are going to make these products benefit from these products.

	**Emerging South (3/8):** Brazil U, Chile R+U, Indonesia R<br><br>**Developing South (1/3)**: Dominican Republic U	
Processes: Field tests and pilots assess costs, technical feasibility and safety/impacts	**North (3/11)**: Poland R, Sweden R, US R<br><br>**Emerging South (5/8):** Brazil U, Chile U, Indonesia R, Saudi Arabia U, Turkey R<br><br>**Developing South (2/3)**: Dominican Republic R, Kenya U	**Saudi Arabia, Urban:** (All CDR approaches) need accurate experiments under certain conditions… thus it needs a research study at first, before the doing of the experiment. Finance and coordination are the responsibility of the authorities, but before any of that you need research proving whether or not it's beneficial on a certain level, and whether the expenses are suitable or not.<br><br>**Kenya, Urban:** (Regarding BECCS) Myself, I would like to say before there's consideration into putting this into a reality, the government should conduct a pilot project where they set up sites out there to analyze the effects, to analyze how effective is this technology before they introduce it to the communities.
Actors, Processes: Role for expert assessment in strengthening public and stakeholder engagement - education, mutual learning, innovation, learning by doing	**North (4/11)**: Germany R, Italy U, Poland U, US R<br><br>**Emerging South (6/8):** Brazil R, Chile R, India U, Saudi Arabia U, South Africa R, Turkey R+U,	**South Africa, Rural:** I personally think this one the lead should be taken by scientists… to advise the state or the policymakers... However, there should be a multidisciplinary teams… and it should go down as well. You know, this matter can be simplified on the ground level: for example, cutting trees, and not wanting to know how to plant a tree. So it goes back to the community, the very same chain. It's everybody's responsibility.
Actors, Processes: Universities and educational institutes as sites of innovation and knowledge dissemination	**North (4/11)**: Australia R+U, Italy U, Poland U, US U<br><br>**Emerging South (4/8):** Brazil R+U, India U, Indonesia U, Saudi Arabia R+U<br><br>**Developing South (1/3)**: Dominican Republic U	**India, Urban:** I think agricultural institutes should take the initiative… they should do some experimental things there: how the land will be more fertile using (these approaches). And they should be practical, and show how it can be implemented in farming lands. So that can be done through their agricultural students, and in the next generation … land will become more fertile, and we can get more crops and more income. Once it is understood by the farmers, they will easily follow the techniques. And it will be good for our environment.<br><br>**Poland, Urban:** I think young people, secondary school, university students. I mean these projects will in fact be implemented when they are adults, so they will have impact on their execution, I think, but of course they should work in consultation with people who have relevant knowledge, with scientists, with local universities. I mean we generally trust local people.
**Industry and innovation**<br>**Governance dimensions**	**National focus group mentions**	**Representative quotes**

Role: Control industry - Mistrust over government-industry collusion		**North (4/11)**: Australia R+U, Germany U, Poland R, Switzerland U<br><br>**Emerging South (3/8):** Brazil U, Chile R+U, Indonesia R<br><br>**Developing South (2/3)**: Dominican Republic R, Kenya U	**Brazil, Urban:** We know that companies, as a reflection of people, don't do many things voluntarily, I believe a law should pass to oblige companies to care more about the environment and storage and capture carbon dioxide, even using financial retributions (sic). I think the first step is passing a law, we don't see companies voluntarily solving this problem, only a few do.<br><br>**Poland, Rural:** … it should be the government to force these corporations to give a part of their profits, I don't know, or just infrastructure, to help recover… this planet is barely surviving because of just such corporations, who make billions in profits and they only take and give nothing in return. And they should be forced – because they are unpunished, because they pay huge bribes to politicians.
Role: Facilitate industry	Rationale: Trust in government-industry collaboration, corporate technical, material, and intellectual resources	**North (1/11)**: Norway U<br><br>**Emerging South (3/8):** China R+U, India R+U, Saudi Arabia R<br><br>**Developing South (1/3)**: Dominican Republic R	**India, Urban:** (On DACCS) I would like … a lot of industry forums to be done, lot of intra activity among participants, maybe a group newly created where in you have these heads of various ministries and various Public Sector Undertakings (Indian state-owned enterprises) and various bodies such as the Confederation of Indian Industry; Tata, Mitra and all the big names in the industries. So, I think you need to get them together and formulate this idea and then it will be taken up. And of course, you will require the government also to be a part.<br><br>**Norway, Urban:** I would like to see Equinor (Norwegian state-owned petroleum company) take the lead, because they have resources and the finances, and they also have the knowledge and the people to start in a way.
	Rationale: Corporate social responsibility	**Emerging South (3/8):** China U, India U, Turkey U<br><br>**Developing South (1/3)**: Nigeria U	**Turkey Urban:** Social responsibility projects can be done by coming together with the public and institutions. You can meet with different producers and exchange ideas with them, and if they have a land, their lands can be used for trial.<br><br>**Nigeria, Urban:** Like we said, agricultural companies, those that use the agricultural produce as raw resources or materials, they should look for a way to give back to the society. At the end of the day they know that there is nothing they can produce without the help of farmers. It will help them and the government can helped them on mechanized farming because some of them have ideas on farming in a large land, but they are using subsistence means of farming.
**Publics**<br>**Governance dimensions**		**National focus group mentions**	**Representative quotes**

Role: Self- and community engagement	**North (9/11)**: Australia R, Austria R+U, Germany U, Italy U, Norway R+U, Poland U, Spain R+U, Switzerland U, UK U<br><br>**Emerging South (8/8)**: Brazil U, Chile R, China R+U, India U, Indonesia R, Saudi Arabia R+U, South Africa R+U, Turkey R+U<br><br>**Developing South(3/3)**: Dominican Republic R+U, Kenya R+U, Nigeria R+U	**Sweden, Rural:** We should not forget that, unfortunately, we are living in a capitalistic society, and we do vote with the wallet. So, we can do really much. We can choose where we buy our products, and where we buy our food, and where we put our money. We can avoid many of the great emitters ourselves. We have some responsibility on us as well.<br><br>**Chile, Urban:** Well, what can I say, we need to support, what else can we do. We can re-plant a tree, we can clean up, We can do actions that help the whole environment. Generate projects with companies that can do it.<br><br>**Brazil, Urban:** A sustainable stamp like "I don't use carbon dioxide" can be good, "I don't use chemical fertilizers" and the consumer prioritizing buying from these producers will encourage the broader use of these techniques by the companies that pollute. This is a way that the population can contribute by directing the companies that pollute into adopting better practices otherwise their products will not be consumed, and their profit will decrease. Everything is connected to the pocket. This would be a way to force the implementation of these practices.
Role: Informational campaigns, educational programs	**North (11/11)**: Australia R, Austria R, Germany U, Italy R+U, Norway R, Poland R+U, Spain R, Sweden U, Switzerland U, UK R, USA R<br><br>**Emerging South (8/8)**: Brazil R, Chile R+U, China R, India R+U, Indonesia U, Saudi Arabia R+U, South Africa U, Turkey R<br><br>**Developing South (3/3)**: Dominican Republic R+U, Kenya R+U, Nigeria R+U	**Nigeria, Urban:** When I was in school… they taught us the disparity between indigenous (local) media and exogenous (external, state or international) media. No matter how enlightened (sic) people are in rural areas, people still believe in indigenous media. You know, we have print media, but most of them are still illiterate… but that does not mean they do not have the intellect, some of them are even wiser than the graduates. So it is a very nice way to communicate with them, sensitizing them in a language they understand.
Role: Consult local stakeholders	**North (7/11)**: Australia R+U, Austria R, Brazil R, Germany U, Italy U, Poland R, UK R<br><br>**Emerging South (5/8)**: India U, Indonesia R+U, Saudi Arabia R, Turkey R	**Brazil, Rural:** I think the debate is also really important. When you have data you can discuss things, even if the results are positive, not all the population will agree with that and these people are important. Why? Because through them a debate can be generated and this debate can lead to the development of the idea, change something, rethink the project. I think it's very important.<br><br>**Germany, Rural:** I always sort of think it is a matter of whether you include the entire population or the affected population. We talked before about the agriculturalists, who of

		**Developing South (2/3)**: Dominican Republic R, Kenya U	course have to be involved because it affects them directly. With direct air capture, the argument was if this big plant is placed in my neighbourhood, I would like to be involved in the decision making. I do think that the affected populations should be included, just to make it functional long-term. It will not be tolerated long-term, if that is just decided behind the backs of the people, who it affects. It remains to be seen whether the entirety of the German population should be involved. However, I do think that the people, who are regionally affected, should be involved.
Context: Environmental individualization	Demonstrative of climate and environmental priorities and action in society (in global North)	**Emerging South (2/8):** Brazil U, South Africa U,<br><br>**Developing South (1/3)**: Dominican Republic U	**Dominican Republic, Urban:** Ok, look, for example, there is a law in France that is being implemented for 2023, where there will be no plastic covers. If you bring that law here to the Dominican Republic and make it drastic it will cause a disaster. Why? Because of cultural education. We are talking about a person from a developed country, educated from a young age to recycle. They already know how to do things, but not here, so all these things that we have with these measures that would be beneficial, if we do not know how to handle them it can harm us, mainly from the authorities. A clear example of mismanagement was the pandemic.
	Preferences for biogenic approaches	**North (2/11)**: Austria R, Switzerland R	**Austria, Urban:** (Biogenic approaches) are something each one of us could help shape as a project in schools, companies, the entire country, the EU and so on. Everyone can take part. It's like reducing plastic usage or eating less meat, everyone can do it.
	Shifts burden to the individual	**North (4/11)**: Austria U, Italy R+U, Sweden R, Switzerland U,<br><br>**Emerging South (3/8):** Brazil R, Chile U, Turkey U	**Brazil, Rural:** I think this is all part of a system. It doesn't matter if I recycle inside the townhouse if after that in the landfill is going to be mixed up again. It doesn't matter that I have plants inside my house if I chopped off one tree in the street. I think awareness is something very important.<br><br>**Sweden, Rural:** … governments can take responsibility on such; it is not maybe every single individual that has to take responsibility. To look at their carbon dioxide budget, or however it would be called, but that there are big instances who do that. Countries that collect it and process it for every one of us… That it can be controlled in larger scale.
**Government**<br>**Governance dimensions**		**National focus group mentions**	**Representative quotes**
Rationale: Systemic coordination across sectors, levels, regions	National and regional advantages, Integrated multilateral portfolio vs. individual country initiatives	**North (5/11)**: Norway R, Poland U, Sweden R, Switzerland U, US U<br><br>**Emerging South (1/8):** China R	**Poland, Urban:** It also seems to me that maybe we should not limit ourselves to introducing one, but act on a larger scale. In some regions of the world restoring and cultivating vegetation would probably work better. In others – storing carbon, in some industrialized countries, because I understand that it requires building plants that would deal with the processing of coal. Besides, I suspect this carbon dioxide must be pressed down old mine shafts, in such places, so probably DACCS and BECCS could not be used everywhere, because it would have to be in former industrial regions, where there are mines, or some oil wells that are out of commission, because carbon dioxide is pressed under the ground to the depth of several kilometers, so maybe different projects

			are more executable in different places, parts of the world. It should be a global rather than local effort, adapting to the local possibilities of those places where these projects could be implemented.
	Systemic oversight to avoid trade-offs, maintain infrastructure	**North (7/11)**: Germany R+U, Italy R+U, Norway R, Poland U, Sweden R+U, USA R, UK R<br><br>**Emerging South (7/8):** Chile R, China U, Indonesia R, Poland U, Saudi Arabia U, South Africa R, Turkey R<br><br>**Developing South (2/3)**: Dominican Republic R, Nigeria U	**China, Urban:** First, it is a topic to be agreed by various countries, and an agreement made. There should be some agreement signed. Then research about the land use in whole countries, what trees to be planted, what soil condition in different environments, and all this preparation work.<br><br>**Norway, Rural:** But there should be an organisation that others have trust in like others have mentioned the UN. Perhaps EU that represent big areas and many countries, because it can't be that one country makes the decision on their own. You should gather people from different areas, and even from the industry that could be affected by like, like farmers, so that everyone gets their say. And even normal people, that they can vote on it.
	Historic responsibility	**North (4/11)**: Germany R, Norway R, Poland R, Switzerland U<br><br>**Emerging South (1/8):** Brazil U	**Brazil, Urban:** I think the scientific community of the developed countries, especially, should make a firm statement because as they are the ones doing more harm, they don't speak much. The biggest devasted areas are in Europe, the U.S., and China, not in Brazil.<br><br>**Germany, Rural:** When it comes to implementing all this technology – I mean, okay, even simple countries could do it with all the plants – but of course, Africa has had much lower CO2 emissions over the last 100 years than the USA or Europe, for example. This CO2 that we have emitted, which is now in the atmosphere – if we then take that out, then the polluters are essentially the ones that clean that up and that is then also a nice thought, in my opinion.
Trust in levels of governance	Rationale: Low trust in national government on diverse issues	**North (3/11)**: Australia R, Italy U, Poland U R<br><br>**Emerging South (2/8):** Chile R, South Africa R	**Spain, Rural:** I don't trust them, honestly. It's all talk. You saw G8, they talked about pollution, and no one does anything. It's always the same. I don't trust them. There have been so many climate summits and nothing changes, because they're not interested because they have their interests. They have to produce, and climate change requires the contrary. Petrol companies complain because if we start using electric cars they won't get profits, it's all the same… So, governments are biased.<br><br>**South Africa, Rural:** How can you be a Minister of Education today, tomorrow a Minister of Agriculture, and tomorrow a Minister of Police? … It's going to be so easy for you guys to adapt, because you guys (referring to global North populations) get to raise your opinions, but (here) the government decides for you in the future… in South Africa we have the biggest problems implementing… like you say, (we're) being led by a blind man.

	Rationale: High trust in national government	**North (2/11)**: Norway U, Sweden U<br><br>**Emerging South (2/8):** China R+U, Saudi Arabia U	**Sweden, Urban:** You could say, you need to go backwards – it is the politicians that are sitting there that need to have a long-term solution. No short-sighted ideas, this is something to be done long-term… There needs to be a delegation of responsibility down to the municipal level.<br><br>**Saudi Arabia, Urban:** The government know what is best for the people, and they're people before anything else, and they work for the general benefit of the people and know what they should do when it comes to vegetation and the health of it and the health of people which are all important stuff so our role as a community is to support them and to keep the crops, environment, and hardware you might install.
	Rationale: High trust in local governance	**North (9/11)**: Australia R+U, Germany U, Italy R+U, Norway R, Poland R, Spain R+U, Sweden R+U, UK R+U, US R+U<br><br>**Emerging South (7/8):** Brazil R+U, China U, India R+U, Indonesia U, Saudi Arabia U, South Africa R, Turkey R+U<br><br>**Developing South (3/3)**: Dominican Republic R, Kenya R, Nigeria U	**Italy, Urban:** I believe that regions as well should have a role. They can even implement supporting policies as it happens. For example in Trentino Alto Adige, people receive incentives to change into green activities. They can distribute incentives taken from the European Community, through channels such as the EU. In order to do things we need money. Besides all the nice words, we know that municipalities do not have money. We should create policies that support such activities.<br><br>**US, Urban:** I don't know how this could be done without government involvement. I don't like government involvement. But I think on something like this, there needs to be, so there'd have to be like some type of guidelines. But then on the local level, you know, you would be able to adapt within the, the rules and guidelines, you know, what works best for your area. So that, you know, you're all working towards the same goal, but different areas might have to tweak things a little bit for, you know, what they have available in that area in that state or, or county.
Multilateral governmental coordination	European Union	**North (3/11)**: Germany U, Poland U, Sweden U	**Germany, Urban:** Especially agriculture is managed by the EU, and you need the means to do it. There are big and small farmers, so we need the EU and the state to support this. We need supranational organisations for this.<br><br>**Poland, Urban:** I personally think that the EU has done a lot of good, but also a lot of bad. I mean we are also very limited by the EU, and we have not much room to show off. The first thing I can think of is a ban on coal mining for us, ignoring the aspects of whether it is eco or not, but this is a huge amount of money for us and instead of having our own energy, we must use energy from other countries.
	UN template – UNFCCC and other familiar antecedents	**North (6/11)**: Australia U, Germany R, Italy R+U, Norway R, Poland R, Sweden R	**Italy, Urban:** In an ideal world, it would be nice to have a scientific committee (referring to but somewhat misunderstanding the UNFCCC) that can make decisions and it can even impose guidelines to the different governments. I don't think it should be done by one government but it should be done by a scientific committee, so that there are no issues between different governments and interests of one or the other prevail over the other. We mentioned China - the main issue related to the environment in China is that

		**Emerging South (5/8):** Brazil U, China R+U, Saudi Arabia R, South Africa R<br><br>**Developing South (2/3)**: Dominican Republic R, Nigeria U	different countries have tried implementing regulations on pollution but it's very difficult… They cannot impose changes because otherwise China will stop exporting several things. The same thing happens in the US, in Italy and in Europe. At a worldwide level, that defines guidelines that are imposed on all the different governments, so that they do not get in conflict with one another.

Supplementary Table 3 is a longer version of Table 3 (in text), with all themes (hopes, concerns and governance), as well as additional quotations from a wider range of focus groups. Here is also included (in the column 'National focus groups mentions'), a count of the focus groups and countries that spoke to a certain theme.
