# Supplementary material for: Public perceptions on carbon removal from focus groups in 22 countries
Source: Nat Commun. 2024 Apr 24;15:3453. doi: 10.1038/s41467-024-47853-w (PMC11043362; doi:10.1038/s41467-024-47853-w)
Supplement: Supplementary file 3 — Reporting Summary [file 41467_2024_47853_MOESM3_ESM.pdf]

nature portfolio

Corresponding author(s): LOW, Sean

Last updated by author(s): 28.02.2024

Reporting Summary

Nature Portfolio wishes to improve the reproducibility of the work that we publish. This form provides structure for consistency and transparency in reporting. For further information on Nature Portfolio policies, see our Editorial Policies and the Editorial Policy Checklist.

Please do not complete any field with "not applicable" or n/a. Refer to the help text for what text to use if an item is not relevant to your study.
For final submission: please carefully check your responses for accuracy; you will not be able to make changes later.

Statistics

For all statistical analyses, confirm that the following items are present in the figure legend, table legend, main text, or Methods section.

n/a	Confirmed	
X	☐	The exact sample size (*n*) for each experimental group/condition, given as a discrete number and unit of measurement
X	☐	A statement on whether measurements were taken from distinct samples or whether the same sample was measured repeatedly
X	☐	The statistical test(s) used AND whether they are one- or two-sided<br>*Only common tests should be described solely by name; describe more complex techniques in the Methods section.*
X	☐	A description of all covariates tested
X	☐	A description of any assumptions or corrections, such as tests of normality and adjustment for multiple comparisons
X	☐	A full description of the statistical parameters including central tendency (e.g. means) or other basic estimates (e.g. regression coefficient) AND variation (e.g. standard deviation) or associated estimates of uncertainty (e.g. confidence intervals)
X	☐	For null hypothesis testing, the test statistic (e.g. *F*, *t*, *r*) with confidence intervals, effect sizes, degrees of freedom and *P* value noted<br>*Give P values as exact values whenever suitable.*
X	☐	For Bayesian analysis, information on the choice of priors and Markov chain Monte Carlo settings
X	☐	For hierarchical and complex designs, identification of the appropriate level for tests and full reporting of outcomes
X	☐	Estimates of effect sizes (e.g. Cohen's *d*, Pearson's *r*), indicating how they were calculated

Our web collection on statistics for biologists contains articles on many of the points above.

Software and code

Policy information about availability of computer code

Data collection	Zoom (version 5.17.7 (31859))
Data analysis	MaxQDA (MAXQDA Standard 2022, Release 22.8.0, (c) 1995-2022 VERBI GmbH Berlin).

For manuscripts utilizing custom algorithms or software that are central to the research but not yet described in published literature, software must be made available to editors and reviewers. We strongly encourage code deposition in a community repository (e.g. GitHub). See the Nature Portfolio guidelines for submitting code & software for further information.

Data

Policy information about availability of data

All manuscripts must include a data availability statement. This statement should provide the following information, where applicable:

- Accession codes, unique identifiers, or web links for publicly available datasets
- A description of any restrictions on data availability
- For clinical datasets or third party data, please ensure that the statement adheres to our policy

The datasets generated during and/or analyzed during the current study are not publicly available at present, to allow for further analysis and publication of findings over the course of our multi-year project (European Research Council Grant Agreement No. 951542-GENIE-ERC-2020-SyG). Access to the raw data can be made available from the corresponding author on reasonable request after an embargo of two years, during which time our analysis continues. The totality of the dataset will be made publicly available in full following the conclusion of the GENIE project.
A minimum supporting dataset that is necessary to interpret, verify and extend the research in the article – narrowed from 44 focus group transcripts – is supplied through Supplementary Tables 1-3.

Research involving human participants, their data, or biological material

Policy information about studies with human participants or human data. See also policy information about sex, gender (identity/presentation), and sexual orientation and race, ethnicity and racism.

Reporting on sex and gender	Sex and gender are not considered as part of the analysis of this paper.
Reporting on race, ethnicity, or other socially relevant groupings	Race and ethnicity were not considered as part of the analysis of this paper.
Population characteristics	Focus groups were screened for an even split between genders, between 18-44 yrs and 45+ yrs cohorts, division between urban and rural, and for distribution across education level, income, occupation type, and region.
Recruitment	Participants self-defined whether they are climate deniers. Researchers judged this necessary to maintain topical targetedness, as well as goodwill. Participants were screened for an urban (including suburban) or rural background, which was self-defined. These determined if participants might be sorted into Urban or Rural focus groups - but differences between these were not parsed in the present study and play no role in influencing results.
Ethics oversight	All components of the research were granted ethical approval by the Reseach Ethics Committee of Aarhus University (#2021-13)

Note that full information on the approval of the study protocol must also be provided in the manuscript.

Field-specific reporting

Please select the one below that is the best fit for your research. If you are not sure, read the appropriate sections before making your selection.

☐ Life sciences ☑ Behavioural & social sciences ☐ Ecological, evolutionary & environmental sciences

For a reference copy of the document with all sections, see nature.com/documents/nr-reporting-summary-flat.pdf

Life sciences study design

All studies must disclose on these points even when the disclosure is negative.

Sample size	
Data exclusions	
Replication	
Randomization	
Blinding	

Behavioural & social sciences study design

All studies must disclose on these points even when the disclosure is negative.

Study description	Qualitative study deploying 44 focus groups in 22 countries to gauge public perceptions on carbon removal technologies
Research sample	Focus groups were not representive of national populations. Sampling was structured to provide a relatively even spread across population characterics, to foster inclusion and representation across gender, education level, income, occupation type and region.
Sampling strategy	See below, due to space constraints.
Data collection	Data was collected by recording over Zoom, and transcribed by Norstat.
Timing	Data collection proceeded from August to December 2022.
Data exclusions	No data was excluded.
Non-participation	No participants dropped out or declined participation.
Randomization	Participants were randomized to the extent that sampling was based on probability sampling and the presence of certain splits and representations of gender, age, locality, education, income, occupation type, and region.

Sampling strategy: Focus groups sizes aimed at 5-8 participants; standard practice calls for less than 10 in a deliberative engagement. Probability sampling was used, with screening for based on an even split between genders, between 18-44 yrs and 45+ yrs cohorts, division between urban and rural; and for distribution across education level, income brackets, occupation type, and sub-national regions unique to each country. Data saturation was not considered - focus group number and locations were determined beforehand, and were conducted and analyzed to completion.Researchers were not blinded to experimental condition and/or the study hypothesis.

Ecological, evolutionary & environmental sciences study design

All studies must disclose on these points even when the disclosure is negative.

Study description	
Research sample	
Sampling strategy	
Data collection	
Timing and spatial scale	
Data exclusions	
Reproducibility	
Randomization	
Blinding	

Did the study involve field work? ☐ Yes ☐ No

Field work, collection and transport

Field conditions	
Location	
Access & import/export	
Disturbance	

Reporting for specific materials, systems and methods

We require information from authors about some types of materials, experimental systems and methods used in many studies. Here, indicate whether each material, system or method listed is relevant to your study. If you are not sure if a list item applies to your research, read the appropriate section before selecting a response.

Materials & experimental systems

n/a	Involved in the study	
X	☐	Antibodies
X	☐	Eukaryotic cell lines
X	☐	Palaeontology and archaeology
X	☐	Animals and other organisms
X	☐	Clinical data
X	☐	Dual use research of concern
X	☐	Plants

Methods

n/a	Involved in the study	
X	☐	ChIP-seq
X	☐	Flow cytometry
X	☐	MRI-based neuroimaging

Antibodies

Antibodies used	
Validation	

Eukaryotic cell lines

Policy information about cell lines and Sex and Gender in Research

Cell line source(s)

Authentication

Mycoplasma contamination

Commonly misidentified lines
(See ICLAC register)

Palaeontology and Archaeology

Specimen provenance

Specimen deposition

Dating methods

☐ Tick this box to confirm that the raw and calibrated dates are available in the paper or in Supplementary Information.

Ethics oversight

Note that full information on the approval of the study protocol must also be provided in the manuscript.

Animals and other research organisms

Policy information about studies involving animals; ARRIVE guidelines recommended for reporting animal research, and Sex and Gender in Research

Laboratory animals

Wild animals

Reporting on sex

Field-collected samples

Ethics oversight

Note that full information on the approval of the study protocol must also be provided in the manuscript.

Clinical data

Policy information about clinical studies

All manuscripts should comply with the ICMJE guidelines for publication of clinical research and a completed CONSORT checklist must be included with all submissions.

Clinical trial registration

Study protocol

Data collection

Outcomes

Dual use research of concern

Policy information about dual use research of concern

Hazards

Could the accidental, deliberate or reckless misuse of agents or technologies generated in the work, or the application of information presented in the manuscript, pose a threat to:

No	Yes	
X	☐	Public health
X	☐	National security
X	☐	Crops and/or livestock
X	☐	Ecosystems
X	☐	Any other significant area

Experiments of concern

Does the work involve any of these experiments of concern:

No	Yes	
X	☐	Demonstrate how to render a vaccine ineffective
X	☐	Confer resistance to therapeutically useful antibiotics or antiviral agents
X	☐	Enhance the virulence of a pathogen or render a nonpathogen virulent
X	☐	Increase transmissibility of a pathogen
X	☐	Alter the host range of a pathogen
X	☐	Enable evasion of diagnostic/detection modalities
X	☐	Enable the weaponization of a biological agent or toxin
X	☐	Any other potentially harmful combination of experiments and agents

Plants

Seed stocks

Novel plant genotypes

Authentication

ChIP-seq

Data deposition

☐ Confirm that both raw and final processed data have been deposited in a public database such as GEO.

☐ Confirm that you have deposited or provided access to graph files (e.g. BED files) for the called peaks.

Data access links
May remain private before publication.

Files in database submission

Genome browser session
(e.g. UCSC)

Methodology

Replicates

Sequencing depth

Antibodies

Peak calling parameters

Data quality

Software

Flow Cytometry

Plots

Confirm that:

- [ ] The axis labels state the marker and fluorochrome used (e.g. CD4-FITC).
- [ ] The axis scales are clearly visible. Include numbers along axes only for bottom left plot of group (a 'group' is an analysis of identical markers).
- [ ] All plots are contour plots with outliers or pseudocolor plots.
- [ ] A numerical value for number of cells or percentage (with statistics) is provided.

Methodology

Sample preparation

Instrument

Software

Cell population abundance

Gating strategy

- [ ] Tick this box to confirm that a figure exemplifying the gating strategy is provided in the Supplementary Information.

Magnetic resonance imaging

Experimental design

Design type

Design specifications

Behavioral performance measures

Imaging type(s)

Field strength

Sequence & imaging parameters

Area of acquisition

Diffusion MRI ☐ Used ☐ Not used

Preprocessing

Preprocessing software

Normalization

Normalization template

Noise and artifact removal

Volume censoring

Statistical modeling & inference

Model type and settings

Effect(s) tested

Specify type of analysis: ☐ Whole brain ☐ ROI-based ☐ Both

Statistic type for inference

(See Eklund et al. 2016)

Correction

Models & analysis

n/a	Involved in the study
☐	☐ Functional and/or effective connectivity
☐	☐ Graph analysis
☐	☐ Multivariate modeling or predictive analysis

Functional and/or effective connectivity

Graph analysis

Multivariate modeling and predictive analysis

This checklist template is licensed under a Creative Commons Attribution 4.0 International License, which permits use, sharing, adaptation, distribution and reproduction in any medium or format, as long as you give appropriate credit to the original author(s) and the source, provide a link to the Creative Commons license, and indicate if changes were made. The images or other third party material in this article are included in the article's Creative Commons license, unless indicated otherwise in a credit line to the material. If material is not included in the article's Creative Commons license and your intended use is not permitted by statutory regulation or exceeds the permitted use, you will need to obtain permission directly from the copyright holder. To view a copy of this license, visit http://creativecommons.org/licenses/by/4.0/
